# Potential for Dose Reduction in CT-Derived Left Ventricular Ejection Fraction: A Simulation Study

**DOI:** 10.3390/tomography9060164

**Published:** 2023-11-15

**Authors:** Martin Weber Kusk, Søren Hess, Oke Gerke, Shane J. Foley

**Affiliations:** 1 Radiography & Diagnostic Imaging, School of Medicine, University College Dublin, Dublin 4 Belfield, Ireland; shane.foley@ucd.ie; 2 IRIS—Imaging Research Initiative Southwest, Department of Radiology & Nuclear Medicine, Esbjerg University Hospital, 6700 Esbjerg, Denmark; soren.hess@rsyd.dk; 3 Department of Regional Health Research, Faculty of Health Sciences, University of Southern Denmark, 5230 Odense M, Denmark; 4 Department of Nuclear Medicine, Odense University Hospital, 5000 Odense, Denmark; 5 Department of Clinical Research, University of Southern Denmark, 5000 Odense, Denmark

**Keywords:** computed tomography, functional imaging, ejection fraction, systolic function, dose reduction, simulation, cardiac

## Abstract

Background: Measuring left ventricular ejection fraction (LVEF) is important for detecting heart failure, e.g., in treatment with potentially cardiotoxic chemotherapy. MRI is considered the reference standard for LVEF, but availability may be limited and claustrophobia or metal implants still present challenges. CT has been shown to be accurate and would be advantageous, as LVEF could be measured in conjunction with routine chest–abdomen–pelvis oncology CT. However, the use of CT is not recommended due to the excessive radiation dose. This study aimed to explore the potential for dose reduction using simulation. Using an anthropomorphic heart phantom scanned at 13 dose levels, a noise simulation algorithm was developed to introduce controlled Poisson noise. Filtered backprojection parameters were iteratively tested to minimise differences in myocardium-to-ventricle contrast/noise ratio, as well as structural similarity index (SSIM) differences between real and simulated images at all dose levels. Fifty-one clinical CT coronary angiographies, scanned with full dose through end-systolic and -diastolic phases, were located retrospectively. Using the developed algorithm, noise was introduced corresponding to 25, 10, 5 and 2% of the original dose level. LVEF was measured using clinical software (Syngo.via VB50) with papillary muscles in and excluded from the LV volume. At each dose level, LVEF was compared to the 100% dose level, using Bland–Altman analysis. The effective dose was calculated from DLP using a conversion factor of 0.026 mSv/mGycm. Results: In the clinical images, mean CTDIvol and DLP were 47.1 mGy and 771.9 mGycm, respectively (effective dose 20.0 mSv). Measurements with papillary muscles excluded did not exhibit statistically significant LVEF bias to full-dose images at 25, 10 and 5% simulated dose. At 2% dose, a significant bias of 4.4% was found. With papillary muscles included, small but significant biases were found at all simulated dose levels. Conclusion: Provided that measurements are performed with papillary muscles excluded from the LV volume, the dose can be reduced by a factor of 20 without significantly affecting LVEF measurements. This corresponds to an effective dose of 1 mSv. CT can potentially be used for LVEF measurement with minimal excessive radiation.

## 1. Introduction

Monitoring cardiotoxic effects of certain antineoplastic drugs (e.g., anthracyclines, Trastuzumab) requires regular imaging to measure the left ventricular ejection fraction (LVEF) [1]. LVEF is calculated from the end-diastolic (ED) and end-systolic (ES) volumes (EDV/ESV) of the left ventricle (LV), as measured by various imaging modalities. A decrease in LVEF can indicate drug-induced heart failure, leading to changes in patient management, making accurate measurements essential. MRI is considered the reference standard for LVEF but is limited by cost, availability and contraindications (claustrophobia, implants, etc.). Echocardiography is commonly used, but concerns about reproducibility and operator dependency mean that Multi-Gated Nuclear ventriculography (MUGA) is still used to monitor cardiotoxic side effects. While being considered highly reproducible and well tolerated, MUGA subjects patients to effective doses from 3.5 to 7 mSv per examination [2]. CT-derived LVEF has been shown to correlate well with MRI [3,4]. As most cancer patients undergo regular contrast-enhanced chest–abdomen–pelvis CT, exploiting the contrast bolus to measure LVEF within the same examination should be possible. Such a one-stop protocol can potentially be more cost-effective than using different imaging modalities while reducing patient visits and simplifying inter-departmental coordination. However, to correctly identify ED and ES phases requires that radiation is switched on during the entire cardiac cycle, as opposed to coronary CT angiography (CCTA), where ECG-based radiation pulsing limits the dose to the temporal window of minimum coronary motion. To acquire data from multiple cardiac cycles, retrospectively gated helical scanning with low pitch is necessary, and doses can approach 20–30 mSv [5], with standard CCTA parameters.

To be acceptable in clinical practice, a CT-LVEF protocol must be designed to keep the radiation dose as low as reasonably achievable (ALARA) without compromising the accuracy of LVEF measurements. Several studies have described relatively low-dose functional CT protocols [6,7,8,9] for LVEF measurement. These were performed on relatively small cohorts, and where a reference standard was employed, results were compared to other modalities. Even with high correlation, systematic inter-modality differences have been demonstrated, and serial LVEF values are recommended to be measured using a single modality [10]. Automatic or semiautomatic LVEF measurement software is used routinely to minimise measurement variability and processing time [11]. The LV volume is segmented based on the attenuation difference between contrast-filled LV and myocardium, with the mitral valve plane either manually delineated or automatically detected. In CT, an inverse correlation exists between the applied radiation dose and the standard deviation (SD) of Hounsfield values (image noise). CT vs. MRI LVEF correlation can be improved with increasing contrast-to-noise ratio (CNR) [12]. We did not find any studies comparing high- and low-dose functional CT and the intra-modality effect on LVEF measurements caused by increased image noise. Such a study is ethically problematic due to the need for repeated radiation exposures. Instead, dose reduction may be simulated by introducing image noise to high-dose scans.

The purpose of this study is, therefore, to use noise simulation on functional cardiac CT series to identify how much noise can be reduced without significant LVEF bias compared to full-dose images.

## 2. Materials and Methods

### 2.1. Simulation Data

We retrospectively identified and retrieved studies from the local PACS system (IDS7, Sectra AB, Linköping, Sweden) at the Department of Radiology & Nuclear Medicine, Hospital of Southwest Jutland, according to the criteria below. All patient-sensitive information was removed before further processing. Images were acquired on Dual-Source cardiac CT scanners (Somatom FLASH or FORCE, Siemens Healthineers, Erlangen, Germany) between October 2009 and January 2021.

The inclusion criteria were as follows:

- Helical CCTAs performed without ECG-based dose reduction, including ED and ES phases.
- Maximum phase interval equal to or less than 10% of the cardiac cycle.

The exclusion criteria were as follows:

- LV contrast attenuation below 250 HU, defined as minimally acceptable by SCCT guidelines [13]. ROI measurement was performed on a single axial slice in the ED phase, midway between the mitral valve and apex.
- Severe cardiac motion or respiratory artefacts with distortion of endocardial contours.
- Metal implant artefacts, e.g., from pacemaker leads, metallic aortic valves, or thoracic spine implants.
- Excessive noise. Studies were excluded if the PACS report noted compromised image quality related to noise. Otherwise, the study was evaluated subjectively by the first author, who has over 17 years of experience in cardiac CT.
- Failure to reliably identify ED and ES phases in the time–volume curves as clearly distinguishable maximum and minimum volumes.

Slice thickness and increment were 0.6/0.4 mm, and images were reconstructed at kernel Br32 or Bv40 with an Advanced Model-Based Iterative Reconstruction (ADMIRE) level of 3 and 5, respectively.

### 2.2. Optimisation of Simulation Parameters

All image processing was performed in MATLAB R2021a (MathWorks Inc., Natick, MA, USA). Furthermore, the “TIGRE” plugin was installed [14], in which a function called “addCTnoise” can insert realistic Poisson noise in the sinogram domain by controlling a “photon flux” parameter, linearly correlated with dose. Using retrospective studies, for which raw data were not available, this required transforming images to sinogram space using the Radon transform (native to MATLAB image processing toolbox). Processing was performed on a standard desktop PC with a 3.2 GHz processor, 16 GB RAM and an Nvidia Geforce 1050Ti 4 GB GPU.

We followed the approach demonstrated by Pelt and Batenburg [15] of approximating iterative reconstructions with filtered backprojection (FBP) to keep computational load and reconstruction time within reasonable limits, as each dataset consisted of approximately 2500 to 4000 images. Instead of deriving customised filter functions, we used iterative testing of native reconstruction parameters to determine the combinations yielding the highest correspondence

We used an anatomically and radiologically accurate 3D-printed heart phantom with contrast-filled left heart chambers. The production and validation of using the phantom for automated LVEF measurements was described in a previous paper [16]. This phantom was placed inside an anthropomorphic chest phantom (LungMan N1, Kyoto Kagaku, Kyoto, Japan), and a series of scans were performed on a Siemens Somatom FORCE CT scanner (Siemens Healthineers, Forchheim, Germany). Using a synthetic ECG at 60 BPM, Dual Source, retrospectively gated helical was performed at 13 dose levels, with scan and reconstruction parameters listed in Table 1.

The two kernels were identical to those encountered in the clinical dataset.

**Figure 1:**
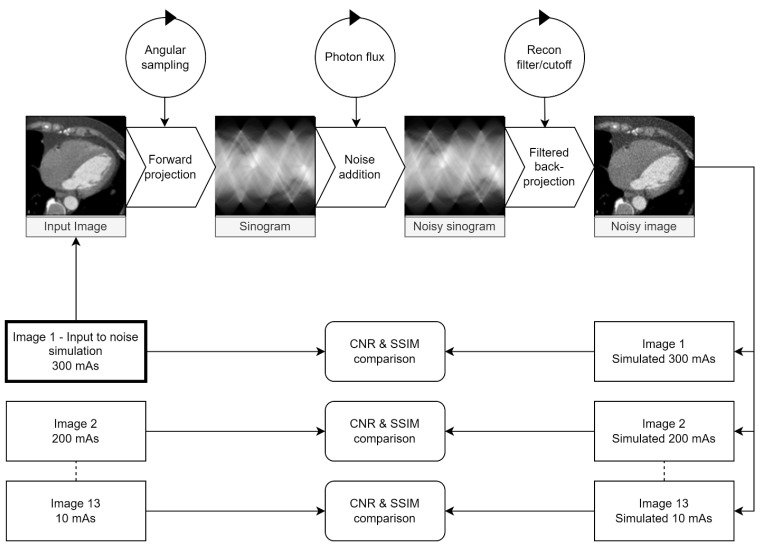
Visualisation of the testing loop used to identify parameters for the maximum similarity between the real and simulated images. Circles represent parameters adjusted at each step. The maximum dose (300 mAs) image was used as input for 13 simulated dose levels for each parameter combination. The contrast-to-noise ratio (CNR) between the myocardium and LV cavity and the SSIM between real and simulated images were calculated and compared for each dose level.

All possible combinations of parameters listed in Table 2 were tested. For each parameter combination, the full-dose (300 mAs) image was used as the simulation input for 13 simulated dose-reduction images corresponding to the physically dose-reduced images. Images were transformed to sinogram space at differing angular resolutions, sampled over 180 degrees. For each simulated dose level, the maximum photon flux value was multiplied by the relative dose reduction, e.g., 20 mAs/300 mAs = 0.07 for the simulated 20 mAs images for a simulated dose reduction to 7% of the original value. After noise insertion, the images were reconstructed with FPB with a combination of reconstruction filter and associated cutoff frequency. All real/simulated image pairs were compared using the metrics described below. The entire process is visualised in Figure 1.

**Figure 2:**
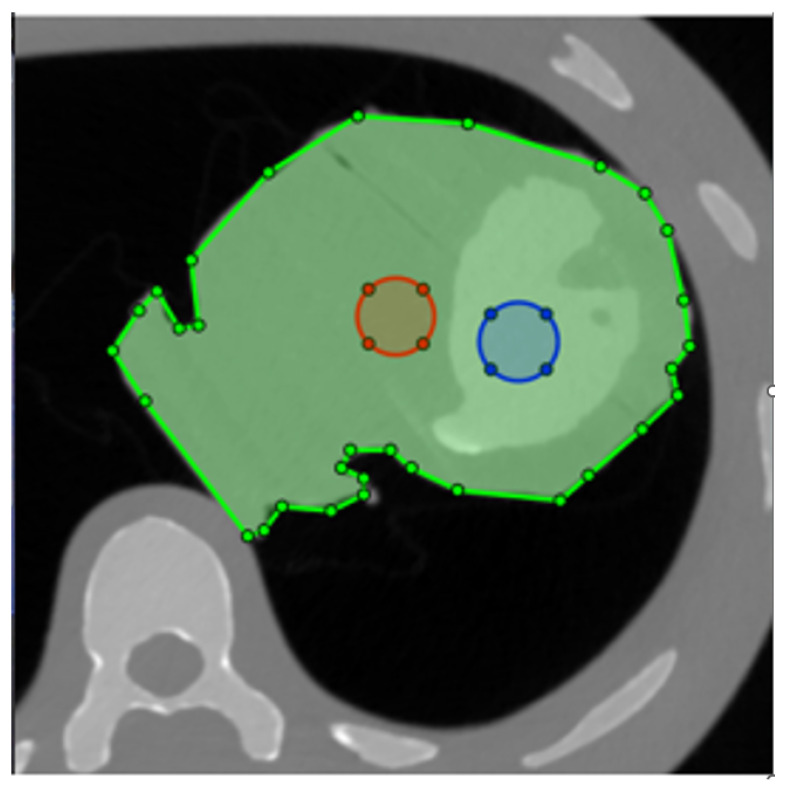
Placement of the two ROIs for contrast-to-noise ratio (CNR) calculation. The phantom is masked with the green ROI, ensuring that the structural similarity index (SSIM) is only calculated for the heart. Blue and red circles are the ROIs used to measure attenuation and noise in the LV and myocardium, respectively.

As LV-to-myocardium differentiation relies on differences in Hounsfield Units (HU) between myocardium and contrast, we calculated the CNR between the phantom LV cavity and the myocardium, whereby two circular ROIs with a diameter of 2 cm were drawn in the phantom LV cavity and “myocardium” as shown in Figure 2. Myocardium-to-LV CNR was calculated as shown in Equation (1) [17]. For the non-simulated images, these values were, of course, constant within the same dose level. The ROIs were automatically propagated to all tested images, eliminating placement variability.

(1) $$\mathrm{CNR}=\frac{\left|\mathrm{HU}(X)-\mathrm{HU}(Y)\right|}{\frac{\left(\mathrm{SD}(X)+\mathrm{SD}(Y)\right)}{2}}=2\cdot \frac{\left|\mathrm{HU}(X)-\mathrm{HU}(Y)\right|}{\left(\mathrm{SD}(X)+\mathrm{SD}(Y)\right)}$$

For each tested parameter combination, linear regression was performed for all 13 dose levels between CNR in the real and simulated images. The slope and R^2^ value were calculated in each case. A slope of one is not necessarily predictive of CNR similarity if individual measurements are scattered about the prediction line, resulting in low R^2^. Likewise, a perfect R^2^ of one but a slope significantly different from one would mean systematic over- or underestimation of simulated CNR.

**Figure 3:**
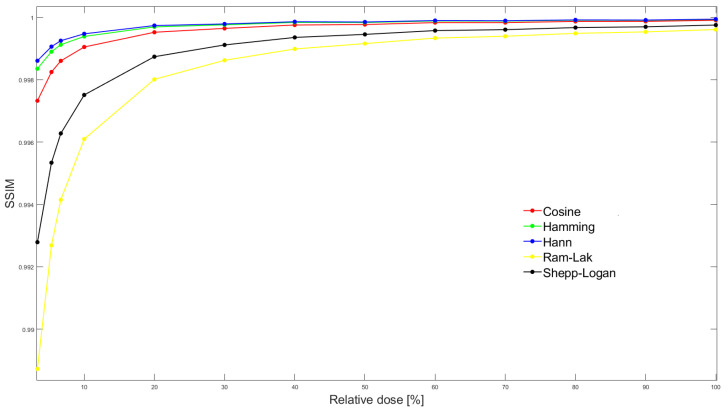
SSIM between simulated and real exposure images at all 13 dose levels. In this particular case, stratified by reconstruction filter, but for fixed values of sampling interval (0.6 degrees), default photon flux (10^5^) and filter frequency cutoff (80%).The area under the SSIM vs dose curve (AUC_SSIM_) is then calculated by integrating SSIM over the entire dose range for each parameter combination.

To optimise noise structure similarity, we calculated the structural similarity index (SSIM) [18] between all pairs of real/simulated images. Using trapezoidal integration, the area under the SSIM vs. relative dose curve (AUC_SSIM_) was calculated for each parameter combination (Figure 3). We masked the phantom contours with a polygonal ROI and thereby excluded other phantom tissue from SSIM calculation (Figure 2).

Due to the high (16-bit) dynamic range inherent in CT images, the SSIM differences are minimal but nevertheless significant. To bring all values to the same scale, slope and coefficient of determination (R^2^) from CNR linear regression of simulated vs. real dose-reduced images and AUC_SSIM_ were normalised to a scale from zero to one. A measure of deviation between real and simulated images was defined from these three metrics. This was defined as the vector length (norm) of the three components. It is shown in Equation (2), where the subscript “norm” indicates normalised values.

(2) $$Deviation=\sqrt{{\left(1-|slope\right|}_{\mathrm{norm}}{)}^{2}+{\left(1-R_{\mathrm{norm}}^{2}\right)}^{2}+{\left(1-AUC_{\mathrm{SSIM},\mathrm{norm}}\right)}^{2}}$$

By finding the minimum of this vector for all parameter combinations tested, the combination of parameters giving the smallest deviation across all three metrics could be located.

### 2.3. Image Processing

A MATLAB R2021a script was used to batch-process all images by slice position and cardiac phase.

The baseline noise level varied from study to study. Therefore, the 100% photon flux level was identified individually by testing 200 initial values between 10^3^ and 10^7^. A circular ROI was drawn in the LV cavity, avoiding PM, while a polygonal ROI was drawn on the lateral myocardium, avoiding visible coronaries. The image was then processed at all 200 initial flux values. ROI SD was measured automatically at all levels, and noise from the corresponding image without noise insertion was subtracted. The data were smoothed using a Gaussian kernel and the minimal difference for each ROI was automatically identified. The mean of the corresponding flux values was then passed to the noise insertion algorithm, and the study reconstructed at five dose levels: 100, 25, 10, 5 and 2% relative dose.

Using a sliding average, output images were reconstructed at 1.0/0.8 mm images and a matrix size of 256 × 256 pixels. This combination has been demonstrated as a good compromise between data volume and volume measurement accuracy [5].

### 2.4. Image Analysis

A single reader measured the ESV, EDV and LVEF using the Syngo.Via Cardiac Function VB50 (Siemens Healthineers, Erlangen, Germany). Measurements were performed both in the standard mode (ST), with papillary muscles (PM) included in the LV volume, as well as the threshold-based “Blood Volume” (BV) mode, excluding PM but including the left ventricular outflow tract (LVOT). Automated measurements were used without manual corrections.

Volumetric CT dose index (CTDI_vol_) and dose-length product were recorded. Effective dose was calculated using a conversion factor of 0.026 mSv/mGycm [19].

### 2.5. Statistics

STATA 17BE (StataCorp, College Station, TX, USA) was used for all statistical analyses, with a significance level of 5%. Bland–Altman analysis was used to calculate mean LVEF bias and 95% limits-of-agreement of the simulated dose-reduced images to 100% dose images. The 95% confidence intervals (CI) of the point estimates were also calculated. The paired *t*-test was used to test for statistically significant bias.

## 3. Results

**Figure 4:**
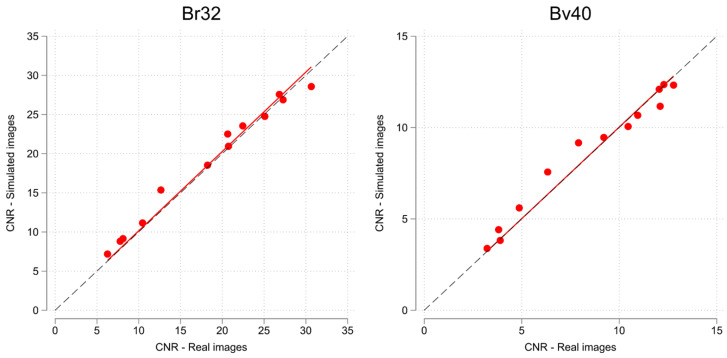
Scatterplot of phantom myocardium to left ventricle CNR, with linear regression fit for all 13 real/simulated dose levels. Note scale differences due to different noise characteristics of the two kernels (Br32 and Bv40). The dashed line represents the line of perfect correspondence.

Fifty-one suitable studies, with a total of 184,847 images, were identified and retrieved. Their characteristics are listed in Table 3. Three patients were scanned at 70 kVp, four at 80, seven at 90, twelve at 100, five at 110, eighteen at 120, and one at 130 and 140 kVp, respectively. The mean LVEF and standard deviation at simulated full-dose level were 65.65% and 10.74% for the BV mode vs. 62.29% and 11.91% for the ST mode, respectively. As per the *t*-test, the difference was significant (*p* < 0.01). The optimised parameters are tabulated in Table 4. As can be appreciated in Figure 4, LV-to-myocardium CNR correspondence between the real and simulated images was high across the entire dose range.

**Figure 5:**
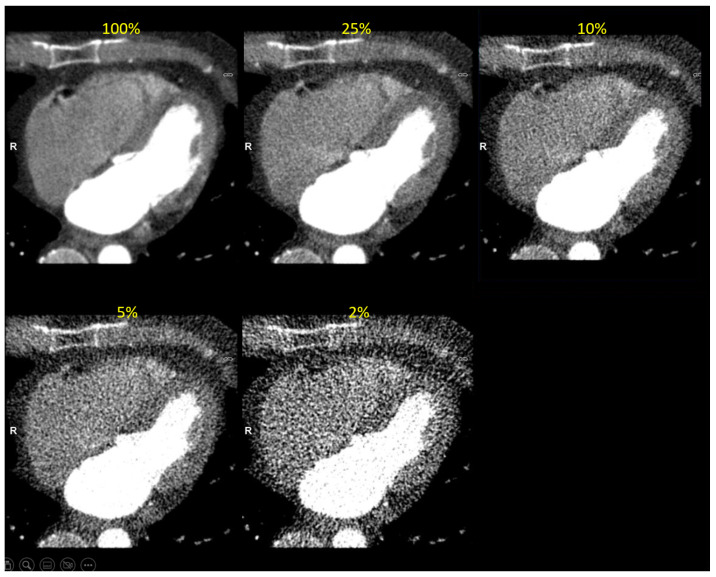
Clinical image reconstructed at five simulated dose levels (in end-diastolic phase). The simulated dose level is indicated in yellow font.

**Figure 6:**
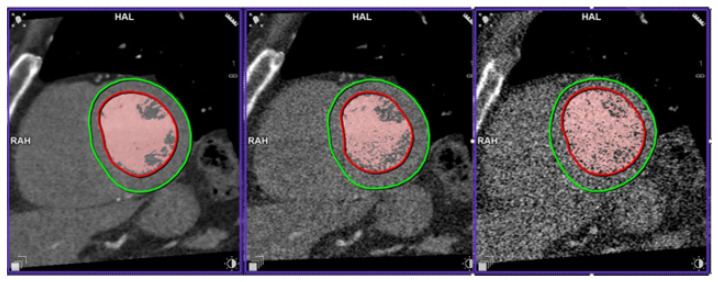
Left ventricle segmentation at 100, 10 and 2% of the initial dose. The red contours are the automatic LV endocardium segmentation. Red-coloured pixels are those above the threshold for LV volume calculation in blood volume mode. The green countours are LV epicardium segmentation, not used in LVEF calculation.

An example of a study simulated at the five dose levels can be seen in Figure 5 and a study with Syngo.Via volume measurement at three dose levels is presented in Figure 6.

**Figure 7:**
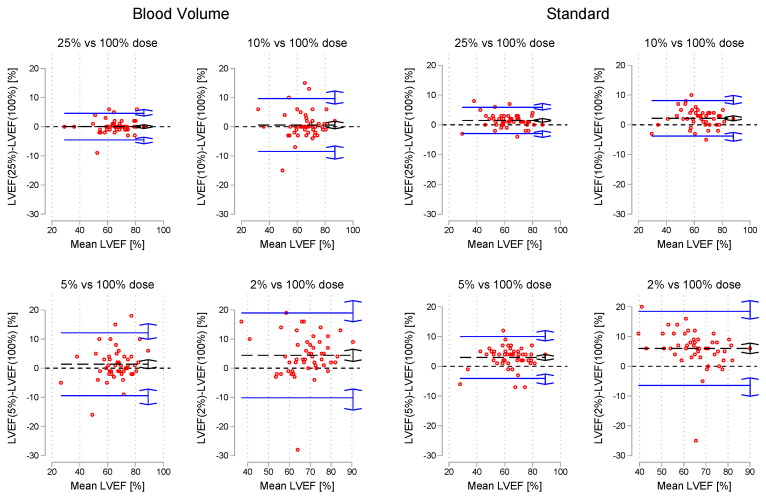
Bland-Altman plots of LVEF for each simulated dose level vs. 100% dose images. The black dashed line is the mean bias, while the blue lines represent the upper and lower limits of agreement. Double arrows are 95% CI of estimates.

Below in Figure 7 and Table 5 are the results of the Bland–Altman analysis of simulated dose levels vs. the 100% dose level images for both measurement modes.

In Table 6, the measured EDV and ESV at dose levels are recorded and statistical differences from the 100% dose level are calculated with the *t*-test.

The mean LV-to-myocardium CNR was 13.6 (6.2) for all included studies at 100% dose. When stratified by low- (100 kVp or below, N = 32) and high-kVp (above 100 kVp, N = 19), CNR was 15.1 (6.2) vs. 11.3 (5.7) with a statistically significant difference (*p* = 0.034). This was caused by a statistically significant difference in attenuation (516 vs. 381 HU, *p* < 0.001), whereas the noise levels were not significantly different (37.4 vs. 31.7 HU in the myocardium ROI, *p* = 0.592). At the 5% dose level, LVEF bias to full-dose images was lower in the low-kVp group for both measurement modes (0.6% vs. 2.6% for the BV mode and 2.5% vs. 3.8% in the standard mode), albeit not significantly so, as 95% CIs overlapped. At that dose level, CNR was 5.3 (2.4) in the low-kVp group vs. 3.6 (1.9) in the high-kVp group. The simulated dose level in the two groups (scaled linearly) corresponded to 26.2 mGycm (0.7 mSv) vs. 59.5 mGycm (1.56 mSv), statistically different at p<0.001.

As mentioned, original images were reconstructed at either kernel Br32 (N = 29) or Bv40 (N = 22). There was no significant differences in 100% dose images of CNR (13.6 (0.9) vs. 13.5 (1.6), *p* = 0.949) or DLP (753.8 (70.0) vs. 795.8 (155.64) mGycm, *p* = 0.791). BV LVEF bias was 0.97% for kernel Br32 vs. 1.91% at kernel Bv40, again with overlapping CIs.

## 4. Discussion

We demonstrated that the simulated dose could be reduced 20-fold, with no significant LVEF bias provided the BV mode was used. By linear scaling, this corresponds to a mean effective dose of 1 mSv (DLP 39 mSv) and across all studies. On the other hand, using the standard mode, there was significant bias caused by progressively larger ESV underestimation with decreasing dose, but measurement uncertainty was less affected by simulated dose reduction. The reasons why the two measurement modes are differently affected may potentially be explained as follows. In the BV mode, all pixels above a certain HU threshold are counted towards the LV volume. Therefore, it is natural that the uncertainty in correct pixel classification is correlated with noise (HU standard deviation), explaining the trend of increasing LoA widths with decreasing doses. The effect can be appreciated in Figure 6. The standard method delineates the LV contours. In image processing, edge detection is preceded by smoothing (e.g., with a Gaussian kernel). Increasing noise can, therefore, result in wider edges, leading to smaller areas, especially in the ES phase, where distinguishing PM from myocardium is more challenging. The difference in absolute LVEF values between the two measurement methods is consistent with that reported by other studies [20] and highlights the importance of using the same method in serial LVEF evaluation. Thus, the choice of measurement method is dependent on whether accuracy or precision is of prime importance.

To our knowledge, this is the first study to systematically explore the effects of dose reduction on functional cardiac images by simulation, using almost one million processed images. Firstly, any confounding effects related to inter-scan and -modality variability were eliminated by using simulation. The approach can be adapted to different scanners and kernels using a simple phantom. Secondly, we identified a potential lower dose limit for reliable LVEF quantification instead of relying on extrapolation from CCTA protocols.

Lee et al. [7] reduced the dose to 4% of CCTA dose using a Somatom FORCE. They found a good correlation to echocardiography at a DLP of 157 mGycm. Lesser et al. [9] achieved a DLP of 95 mGycm in 18 adult patients with congenital heart disease, using a similar approach on a Somatom FLASH, but without comparing functional results to any reference. Even though the scanners used in those studies were similar to those used in the present study, they differed in that the full dose was applied in either ED or ES phases, dependent on heart rate. The studies by Groves et al. [8] and Choi et al. [6] both utilised 320 detector-row CT to deliver DLPs of 88 and 67 mGycm, respectively, but again without comparing volumetric measurements to a reference. It should be noted that effective doses cited in the above-mentioned papers were calculated using somewhat lower conversion factors of 0.014 or 0.017 mSv/mGycm, which should be taken into consideration when comparing results. Nonetheless, our study indicates substantial scope for dose reduction.

Low tube voltages have been demonstrated as an effective tool to reduce doses in contrast-enhanced CCTA [21]. The papers cited above also utilised low-kVp techniques, and our sub-group analysis by high/low tube voltage would seem to support those findings. However, a selection bias towards larger patients is likely present in the high-kVp group. The effect of controlling the photon flux parameter is analogous to adjusting the reference mAs for ATCM. However, when used in conjunction with automated tube voltage selection (ATVS), the tube voltage may be altered, thus affecting contrast attenuation. This effect was not simulated in our study but will have to be considered when designing a clinical protocol. Different ATCM systems behave differently, and when extrapolated from our results, the potential non-linear behaviour at different settings should be accounted for [22]. The choice of kernel did not affect CNR or LVEF bias significantly, even at the same mean dose level. This is probably a consequence of reducing the matrix size below the in-plane spatial resolution limits. Using a 256 × 256 matrix reduces data volume fourfold and still yields a spatial resolution comparable to cardiac MRI at a typical 200 mm cardiac field-of-view. Doubling the slice thickness halves the volume yet again, with associated lower demands on computational power and storage space. We deliberately chose to compare the all dose-reduced series to a 100% dose series simulated at the same kernel-specific reconstruction parameters in Table 4. Thus, for each patient, all images differed only in the level of image noise. It is not unreasonable to expect that using deep learning image reconstruction techniques, image noise and dose may be reduced even further than our study suggests [23].

Our results provide approximated realistic parameter levels for a clinical protocol solely for clinical LVEF measurement with limited scope for coronary diagnostics. DLP is, of course, dependent on scan length and cannot be used as a guideline. Moreover, there may be potential for reducing the scan range only to cover the LV. At the 5% dose level, the scaled CTDI_vol_ was 2.3 mGy, and conservatively rounding up to 2.5 mGy would be our suggestion when programming the ATCM. A clinical protocol should be validated against an external reference such as short-axis cardiac cine MRI, which is complicated by intrinsic modality differences (primarily spatial and temporal resolution) [3,24], hemodynamic effects of contrast media [25] and different measurement software [26]. Ideally, before a clinical study, our simulation could be applied to a dataset of full-dose functional CT images with a corresponding reference modality. Such an approach could help disentangle the effects of the differences mentioned above from that of noise introduced by dose reduction.

Low-pitch, retrospectively gated helical scanning is the most basic of cardiac imaging protocols and can be performed on all scanners equipped with ECG-gating hardware and software, although at potential trade-offs in temporal resolution. Thus, our protocol could potentially be implemented on a standard 64-slice scanner to take advantage of CAP contrast injection for a one-stop protocol at one-fifth of the MUGA dose [2].

### Limitations

A major limitation is the lack of height and weight information in included studies. However, as all included studies were performed using ATCM, the wide dispersion of CTDI_vol_ values indicates a similar diversity in patient sizes. There was a substantial variation of CTDI_vol_ values in the included scans, likely due to different patient characteristics and indications. The range is comparable to that reported by [21] when comparing doses from different hospitals, and it is believed that our cohort can be considered a representative sample of a northern European population.

Only a single software analysis platform was used, with fully automatic measurements and no user corrections. Other software may perform differently [26] and will have to be validated independently. It is theoretically possible that human observers may improve LVEF measurement accuracy by manually delineating contours. Still, it is assumed that this will be highly time-consuming and at risk of perceptual error due to the high noise levels.

The approach to noise simulation was very pragmatic. The focus was on optimising CNR correspondence across the entire dose range. It can be argued that using the phantom to arrive at proposed simulation parameters is not representative of real patients who are not as homogeneous and subject to cardiac and respiratory motion. Using noise and attenuation for comparing simulated dose reduction has been performed before [27] using a similar approach to ours but in a swine model instead of a phantom. Even if this enriched the study with cardiac motion, a limitation was that contrast attenuation was different in simulated and real images.

## 5. Conclusions

Radiation dose can potentially be reduced by a factor of 20 in retrospectively gated cardiac CT to a CTDI_vol_ of 2.5 mGy without significantly affecting LVEF. If results can be validated in a prospective study, CT may be a viable alternative to MUGA for serial monitoring of LVEF, with a large cumulative dose reduction.

## Figures and Tables

**Table 1:** Exposure parameters for 3D printed phantom scanning.

Parameter	Value (s)
Collimation [mm]	2 × 96 × 0.6
Tube voltage [kVp]	120
Tube time–current range [mAs]	10–300
Pitch	0.25
Slice thickness/increment [mm]	1.0/0.8
Matrix	256 × 256
Kernel/ADMIRE level	Br32/5, Bv40/3
Scan range [mm]	160

**Table 2:** Different parameter combinations tested for simulation parameter optimisation. The angular sampling interval is for forward-projecting to sinogram space. Initial photon flux describes the photon flux corresponding to 100% dose. The reconstruction filter and the associated spatial cutoff are equivalent to the FBP reconstruction kernel.

Initial Photon Flux [×10^3^]	Sampling Interval [degrees]	Filters	Cutoff [%]
1	0.4	Ram-Lak	20
5	0.5	Shepp-Logan	40
10	0.6	Cosine	60
50	0.7	Hamming	80
100	0.8	Hann	100
500	0.9		
1000	1.0		
5000			
10,000			

**Table 3:** Characteristics of studies included for noise simulation. Height/Weight was only available in three cases, so it was not tabulated.

Sex [M/F]	Median Age [years] (Min-Max)	Mean DLP [mGycm] (SD)	Mean CTDI [mGy] (SD)	Mean Eff. Dose [mSv] (SD)
27/24	73 (31–92)	771.9 (545.8)	47.1 (31.6)	20.1 (14.7)

**Table 4:** Optimal parameters for both kernels and their metrics as determined in the iterative testing loop.

Original kernel	Br32	Bv40
Filter	Hamming	Hamming
Angular sampling [degrees]	0.6	0.4
Filter cutoff [%]	0.6	0.8
Default photon flux	5 × 10^5^	10^5^
Normalised AUC_SSIM_	0.998	0.994
CNR regression slope	1.011	1.003
CNR R^2^	0.962	0.978

**Table 5:** LVEF bias and upper/limits-of agreement in comparison with 100% dose images. Figures in parentheses are 95% CI of point estimates. *p*-values resulting from paired *t*-test. “BV” and “ST” represent blood volume and standard mode, respectively.

Dose Level	Bias	*p*-Value	LoA−	LoA+
BV				
25%	0.0 (−0.6,0.7)	0.905	−4.5 (−5.9,−3.6)	4.6 (3.7,5.9)
10%	0.6 (−0.7,1.9)	0.368	−8.5 (−11.1,−6.6)	12.2 (9.9,15.3)
5%	1.4 (−0.2,2.9)	0.081	−9.4 (−12.5,−7.2)	12.2 (9.9,15.3)
2%	4.4 (2.3,6.5)	<0.001	−10.2 (−14.4,−7.1)	19.0 (16.0,23.2)
ST				
25%	1.5 (0.8,2.1)	<0.001	−2.9 (−4.2,−2.0)	5.9 (5.0,7.2)
10%	2.2 (1.3,3.0)	<0.001	−3.8 (−5.5,−2.6)	8.1 (6.9,9.9)
5%	3.0 (2.0,4.0)	<0.001	−4.1 (−6.1,−2.6)	10.0 (8.5,12.0)
2%	6.0 (4.2,7.8)	<0.001	−6.5 (−10.1,−3.9)	18.5 (15.9,22.1)

**Table 6:** Mean ESV and EDV for the different simulated dose levels and both measurement methods. Numbers in parentheses are standard deviations. Asterisks indicate that the mean value is significantly different from the 100% dose level (*p* < 0.05).

Dose Level	EDV [mL]		ESV [mL]	
	BV	ST	BV	ST
100%	126.5 (40.0)	151.9 (45.2)	44.3 (22.5)	58.7 (28.7)
25%	126.3 (42.3)	149.4 (45.5)	44.8 (23.0)	56.3 (28.7) *
10%	130.6 (42.3) *	149.9 (47.6)	44.8 (23.2)	54.8 (28.3) *
5%	129.9 (42.3)	147.7 (47.3)	43.5 (23.2)	52.6 (27.4) *
2%	127.6 (46.1)	147.3 (53.0)	37.8 (20.0) *	46.4 (22.7) *

## Data Availability

Image optimisation and processing scripts can be accessed on the projects Open Science Foundation page (https://osf.io/j9m4t/?view_only=59e9c830c9f24c47961b9eb3a272a177, uploaded 26 October 2023), where measurement data can also be found. According to ethics approval conditions, original image data must be deleted after analysis and, therefore, cannot be made publicly available.

## Abbreviations

The following abbreviations are used in this manuscript:

ALARA	As Low as Reasonably Achieveable
ATCM	Automatic Tube Current Modulation
ATVS	Automatic Tube Voltage selection
AUC	Area Under the Curve
CTDI	Computed Tomography Dose Index
CI	Confidence Interval
DLP	Dose–Length Product
ED(V)	End-Diastolic (Volume)
ES(V)	End-Systolic (Volume)
LVEF	Left Ventricular Ejection Fraction
SSIM	Structural Similarity Index
